# Modification of the chemically induced inflammation assay reveals the Janus face of a phenol rich fulvic acid

**DOI:** 10.1038/s41598-022-09782-w

**Published:** 2022-04-07

**Authors:** Thora Lieke, Christian E. W. Steinberg, Thomas Meinelt, Klaus Knopf, Werner Kloas

**Affiliations:** 1 Department of Ecophysiology and Aquaculture, Leibniz-Institute of Freshwater Ecology and Inland Fisheries, 12587 Berlin, Germany; 2 Faculty of Life Sciences, Humboldt University of Berlin, 10115 Berlin, Germany; 3 Faculty of Environmental Science and Engineering, Kunming University of Science and Technology, Kunming, 650500 China

**Keywords:** Inflammation, Environmental sciences

## Abstract

Inflammation is an essential process as a reaction towards infections or wounding. Exposure to hazardous environmental pollutants can lead to chronic inflammations, where the resolving phase is delayed or blocked. Very contradictory studies have been reported on the pro- and anti-inflammatory effects of humic substances (HSs) leading to significant disagreements between researchers. To a certain extent, this can be attributed to the chemical heterogeneity of this group of xenobiotics. Here we show for the first time that pro- and anti-inflammatory effects can occur by one HSs. We adapted an assay that uses green fluorescence-labeled zebrafish larvae and CuSO_4_ to indue an inflammation. In wild-type larvae, exposure to 50 µM CuSO_4_ for 2 h activated the production of reactive oxygen species, which can be monitored with a fluorescence dye (H2DCFDA) and a microplate reader. This allows not only the use of wild-type fish but also a temporal separation of copper exposure and inflammatory substance while retaining the high throughput. This modified assay was then used to evaluate the inflammatory properties of a fulvic acid (FA). We found, that the aromatic structure of the FA protects from inflammation at 5 and 50 mg C/L, while the persistent free radicals enhance the copper-induced inflammation at ≥ 300 mg C/L.

## Introduction

Inflammation is a normal reaction towards harmful stimuli and plays a critical role during infections or wounds. Inflammation follows a biphasic stage starting with the initiation phase, where cells are attracted towards the affected site and release pro-inflammatory signal molecules such as interleukin (IL)-1β, IL-8, tumor necrosis factor alpha (TNF-α), and reactive oxygen species (ROS)^1,2^. These signal molecules recruit more immune cells towards the site of inflammation. In the resolving phase, anti-inflammatory molecules, e.g., IL-10, transforming growth factor β (TGFβ)^3,4^ are released to end the inflammation. In chronic inflammation, the resolving phase is delayed or blocked. This can be caused by diseases, such as Alzheimer's disease or rheumatoid arthritis^5,6^, but also because of allergic reactions or exposure to hazardous environmental pollutants^7,8^.

Humic substances (HSs) are complex compounds mainly occurring from degrading organic material. They represent up to 95% of the dissolved organic matter (DOC) in freshwater aquatic ecosystems^9–11^. Concentrations range normally between 0 and 50 mg C/L but can exceed 250 mg C/L in tropical water bodies^12^. Both, pro- and anti-inflammatory effects of HSs have been reported. HSs reduced the inflammatory effect of hyperglycemia on vascular endothelial cells, decreased edema, skin diseases, and rheumatoid arthritis^13–15^. On the other hand, the generation of excess ROS and oxidative damage has been reported as well, associated for example with the Blackfoot disease^16–18^. HSs have very heterogenic structures depending on the organic raw material and the degradation pathways from which they are formed, which explain the contradicting reports on the inflammatory effects of HSs. We recently showed that exposure to fulvic acid (FA), a subgroup of HSs, significantly increased the ROS concentration in zebrafish larvae at concentrations ≥ 300 mg C/L and caused tissue damage and mortality^19^. However, at medium concentrations, the same FA induced the expression of anti-oxidative genes without having any detrimental effects. We, therefore, hypothesize that the inflammatory properties do not only differ between different HSs but within one HS depending on the concentration.

In vivo studies on inflammation are difficult due to the opacity of the tissue. To bypass this problem, d'Alençon, et al.^20^ developed an inflammation assay using the transgenic zebrafish strains lysC::DsRED2 and BACmpx::GFP. Copper is used to induce damage at the neuromasts along the lateral line and the migration of the fluorescent protein labeled myeloid leucocytes can be monitored in the living larvae. However, the use of genetically modified organisms (GMOs) requires special precautionary measurements (e.g. disinfection procedures, waste inactivation, special training) to ensure environmental and human safety^21,22^. Furthermore, in many countries working with GMOs is regulated and has to be approved by governmental agencies. For their manual assay, each larva has to be fixated individually to record the fluorescence, preventing high throughput screening. The developed automated detection from d'Alençon et al.^20^ allows high throughput screening but requires equipment, such as an automated fluorescence microscope, and a special software to detect and analyse the fluoresence signals. It is furthermore unsuitable for substances that directly interefere with the copper, e.g., by chelating metals or being oxidized, and thereby preventing the inflammation induction by copper. Those include many promising natural therapeutics, such as HSs, curcumin, and theaflavins^23–25^.We hypothesize that the copper-induced inflammation results in the production of ROS. These ROS can be monitored using a fluorescence dye in normal wild-type zebrafish larvae and a microplate reader. This modified assay can then be used to detect the inflammatory properties of natural compounds such as the FA.

## Results and discussion

### Chemically induced inflammatory assay

To determine the anti-inflammatory property of the FA, we developed an assay based on the methods of ROS detection methods from Hermann et al.^26^ and Goody et al.^27^ and the ChIn from d'Alençon, et al.^20^. Copper is used to induce an inflammatory reaction by damaging the neuromast cells at the lateral line and GFP labeled zebrafish strains to monitor infiltration of leukocytes. We showed that the copper induced inflammation also results in the production of ROS, which can be detected by the conversion of H2DCFDA to the fluorescence-emitting DCF.

#### Copper concentration and time of exposure

**Figure 1:**
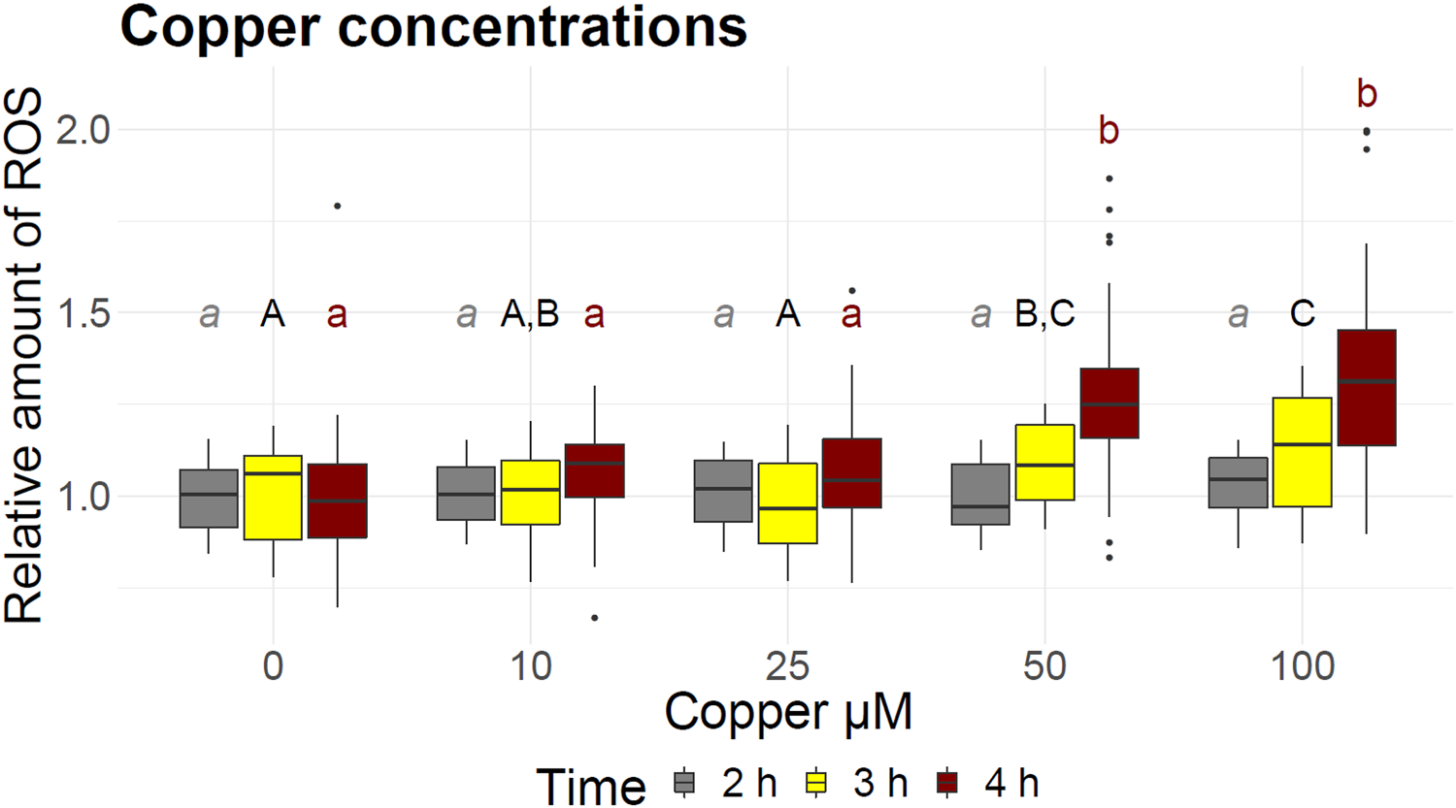
Relative amount of reactive oxygen species (ROS) in larvae exposed to different concentrations of copper. Fluorescence was measured after 2 h, 3 h, and 4 h of incubation with the fluorescence dye. Different letters of the same color represent significant differences within one time-point. ANOVA-HSD, n = 36, df = 4, *p* < 0.05.

After 2 h of incubation with H2DCFDA, none of the copper exposure groups showed any difference in the normalized relative fluorescence units (RFU) compared to the control (Fig. 1). After 3 h of incubation larvae exposed to both, 50 µM and 100 µM copper, produced a significantly increased fluorescence signal (1.1-folds for 50 µM, p = 0.033 and 1.1-folds for 100 µM, p = 0.002). This increase became highly significant (p < 0.001) after 4 h of incubation with the dye (1.2 for 50 µM and 1.3 for 100 µM). Two-way ANOVA revelated a significant effect of the of the copper concentration and the time of incubation with the fluorescence dye (Supplement S3). Posthoc analysis showed a small significant difference between 3 h of incubation compared to 2 h and a high significant different between 4 and 2 h of incubation in the 50 µM and 100 µM group. Neither 10 µM nor 25 µM copper exposure induced a significant increase in ROS production. During the experiments, none of the copper concentrations used resulted in larval mortality. The background fluorescence signal (wells without larvae) was not significant different from the signal of control larvae and both did not change over the time, excluding extra-larval fluorescence of the media.

**Figure 2:**
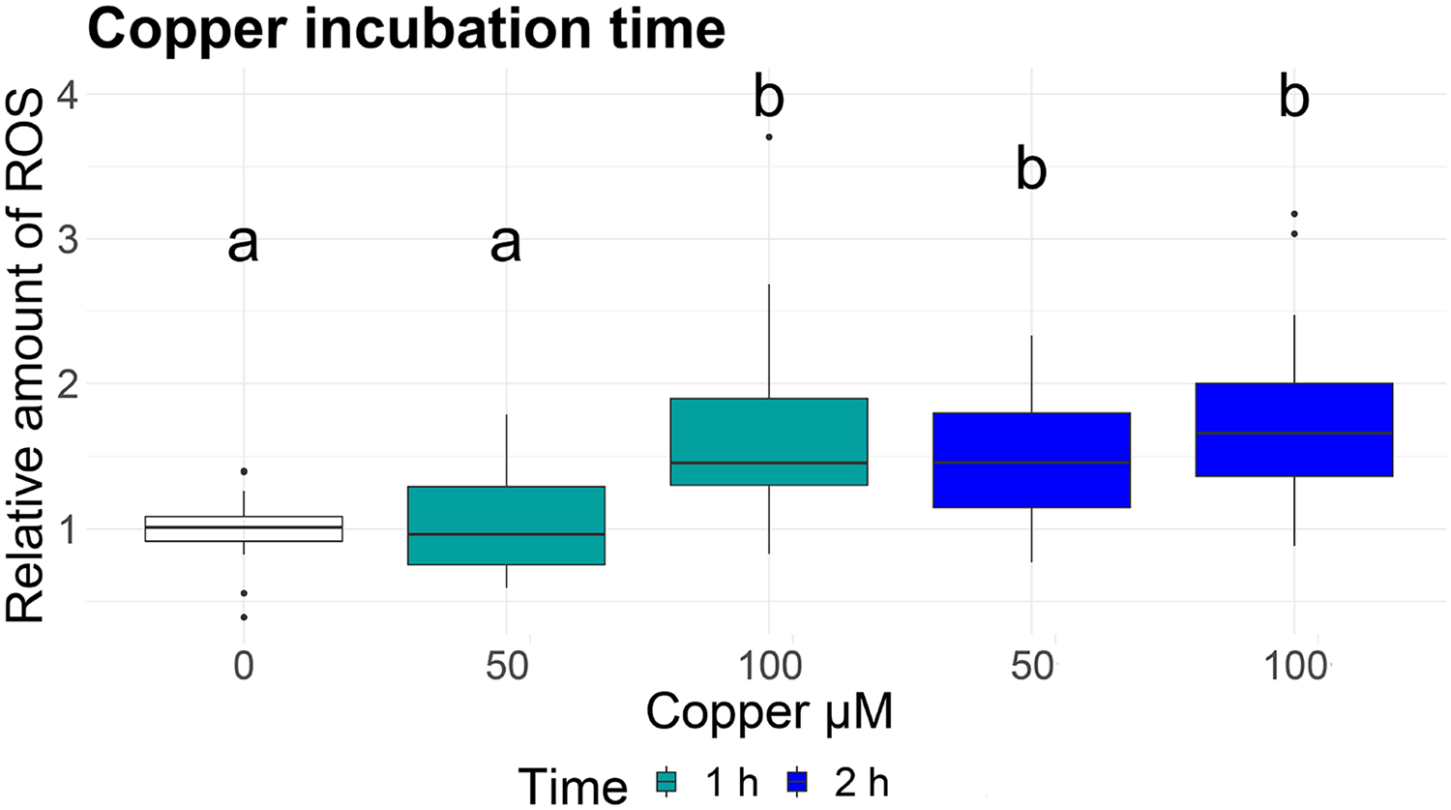
Relative amount of reactive oxygen species (ROS) in larvae exposed to different concentrations of copper for 1 h and 2 h. Fluorescence was measured after 4 h of incubation with the fluorescence dye. Different letters represent significant differences. ANOVA-HSD, n = 36, df = 4, *p* < 0.05.

Exposing larvae for 1 h to 100 µM Cu resulted in a significant increase in ROS production (1.6-fold), while 50 µM Cu did not induce any changes (Fig. 2). After 2 h of incubation, both copper concentrations increased the ROS production significantly, similar to the experiments before. Two-way ANOVA revealed a significant effect of the exposure concentration and the incubation time (Supplement S4). Although no mortality or injuries where visible after exposure to copper, we recommend using 50 µM copper with 2 h of exposure rather than 100 µM. These results differ from those reported by d'Alençon, et al.^20^, who found 40 min exposure with 10 µM CuSO4 to induce the inflammation. Possible reasons are that the 56 hpf larvae used by d'Alençon, et al. 20 might be more sensitive to copper exposure than the 96 hpf larvae used in our experiment. Furthermore, copper toxicity decreases with increasing hardness and alkalinity, and the E3 water used by d'Alençon, et al.^20^ differs from our DIN water in these conditions (E3: 0.33 mM CaCl2 and 0.33 mM MgSO4, DIN water: 2 mM CaCl2 and 0.5 mM MgSO4). Lower concentrations of copper could also be sufficient to recruit the leucocytes to the site of inflammation, while the production of ROS might require higher concentrations. The sensitivity of the larvae might also be specifically to the zebrafish strain used and the individual microbiota composition. Finally, the detection of ROS by fluorescence dye might be less sensitive than the optical measurement of recruitment. Production of ROS might be induced by 10 or 25 µM copper, but with an intensity below the detection limit or in the range of the background signal.

#### Verification of the inflammation assay

To allow the comparison to the assay from d'Alençon, et al.^20^ , the effects of two widespread and commercially available anti-inflammatory drugs on the copper-induced ROS production were analyzed.

**Figure 3:**
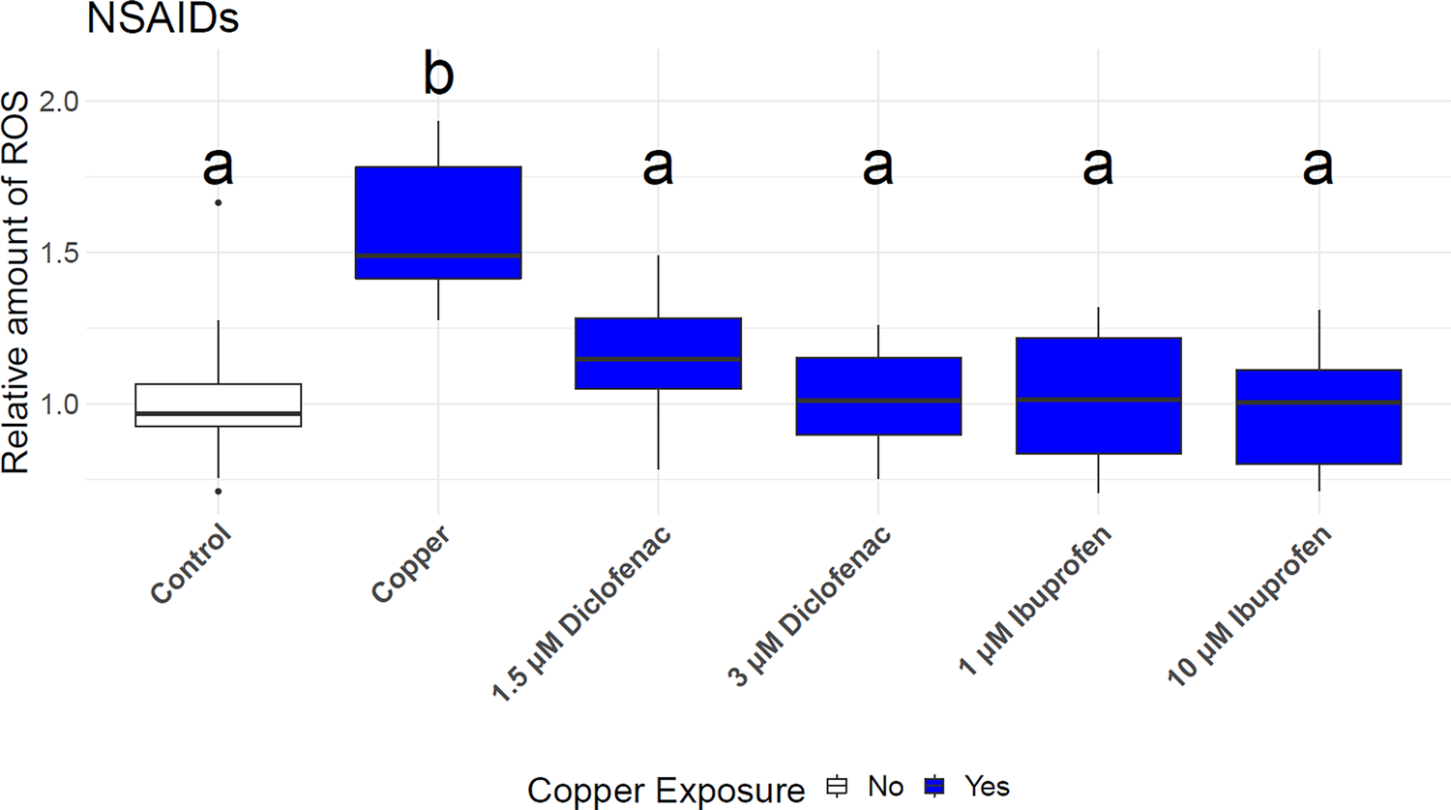
Relative amount of reactive oxygen species (ROS) in larvae treated with diclofenac or ibuprofen before exposed to copper. Fluorescence was measured after 4 h of incubation with the fluorescence dye. Different letters represent significant differences. ANOVA-HSD, n = 24, df = 5, *p* < 0.05.

Exposing larvae to 1.5 and 3 µM diclofenac, and 1 and 10 µM ibuprofen for 1 h before copper exposure, decreased the copper-mediated inflammation significantly (p < 0.01, Fig. 3). There was no significant difference to the unexposed control group.

This supports the findings from d'Alençon, et al.^20^, where the same concentrations of diclofenac and ibuprofen suppressed the copper-induced migration towards the lateral line. Comparing transcriptional changes after ibuprofen and diclofenac exposure, Zhang, et al.^28^ determined that ibuprofen led to higher dysregulation on hemodynamics than diclofenac. Larvae in our assay were exposed only for 1 h to the NSAIDs and 96 hpf are less susceptible than the 50 blastula-stage embryos (2–3 hpf) used to evaluate the effects of diclofenac and ibuprofen. Based on our observation ibuprofen and diclofenac can both be used as positive controls in the assay. Although no significant difference was observed between the two diclofenac concentrations, we decided to use 3 µM diclofenac as a positive control as the trend towards an increased reduction was visible.

The major drawbacks of the live imaging platform by d'Alençon, et al.^20^ are the need for genetically modified zebrafish strains and special equipment and software to allow for high throughput screening. Furthermore, chemicals that interact directly with copper, can not be evaluated with this assay. With our modified assay, wild-type zebrafish larvae and a multiwell plate reader can be used to easily measure the inflammatory properties of chemicals. The temporal separation of exposure to copper and therapeutic of interest furthermore allows to use the assay with antioxidants or higher molecular natural substances which would interfer with copper when applied simultaneously.

Exposing 96 hpf larvae to 50 µM copper for 2 h produced a significant increase in the ROS concentration by damaging the hair cells^29^. Treating larvae for 1 h with ibuprofen or diclofenac before copper exposure diminished the ROS production, showing that the copper induces inflammation and not only exerts oxidative stress. To refine the method, future studies should focus on spacial imaging and validating the source of ROS signal, e.g., by flow cytometry analysis and antibody staining. Nevertheless, our assay can be allready used as an easy to handle alternative or useful addition to the ChIn assay and help to detect the effects of immunomodulatory compounds.

### The inflammatory activity of fulvic acid

**Figure 4:**
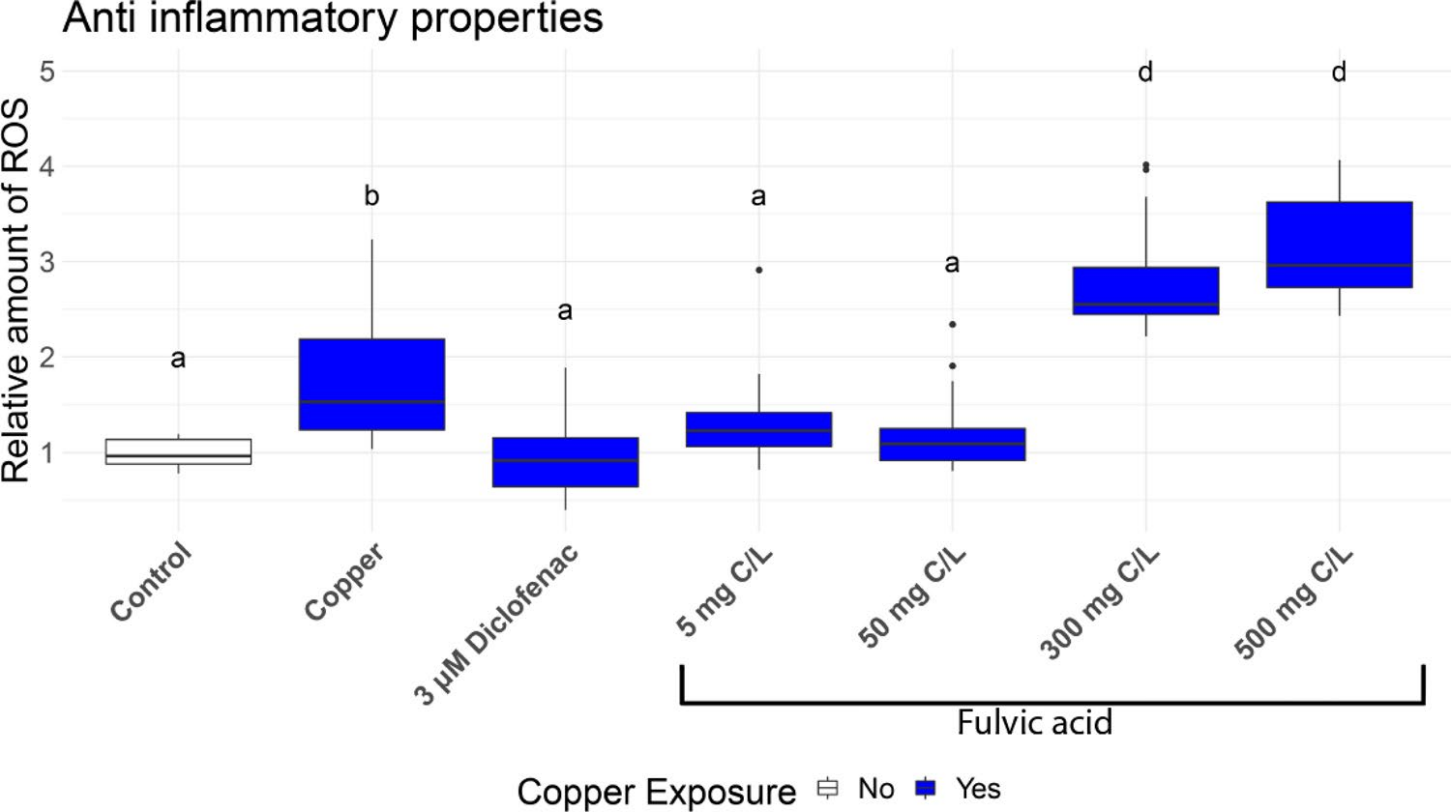
Relative amount of reactive oxygen species (ROS) in larvae exposed to different concentrations of fulvic acid for 96 h and copper for 2 h. Diclofenac larvae were exposed only to diclofenac for 1 h before being exposed to copper. Fluorescence was measured after 4 h of incubation with the fluorescence dye. Different letters represent significant differences. ANOVA-HSD, n = 24, df = 5, *p* < 0.05.

The modified ChIn assay was used to evaluate the inflammatory properties of a FA (Fig. 4). In larvae exposed only to copper without FA conditioning, the ROS concentration increased 1.7 times, compared to the control (no FA, no copper exposure). In contrast, ROS concentrations in the larvae conditioned with 5 mg C/L and the 50 mg C/L FA differed neither significantly from the control without exposure to copper nor from the diclofenac group (1.3 and 1.1 times). However, larvae raised in 300 and 500 mg C/L showed significantly higher ROS concentrations than both control groups and the diclofenac group (2.8 and 3.2 times).

Anti-inflammatory effects of HS have previously been reported in terrestrial vertebrates. Topical application of oxifulvic acid reduced the cutaneous inflammation produced by dinitrofluorobenzene in mice in a comparable way as diclofenac sodium or betamethasone did^30^. In lymphocytes, increased expression of the anti-inflammatory IL-2 was observed after exposure to 20 to 100 mg/L of a potassium humate^31^. The classical and alternative complement pathway and pro-inflammatory cytokines TNF-α, IL-1ß, and IL-6were suppressed by 40 mg/L of the same substance. Contradicting these findings, pro-inflammatory effects and oxidative stress caused by HSs have been reported as well. Exposing juvenile pacu (*Colossoma macropomum*) to 20 to 80 mg C/L of a natural and a commercial HS for 10 days induced the concentration and activity of cytochrome P450 1A (CYP1A), which has been linked to oxidative damage and inflammation^32,33^. These results show that HSs, in general, can do both, exert oxidative stress, leading to tissue damage and inflammation, and protect against oxidative stress. However, as HSs differ structurally greatly from each other, biological results from different HSs cannot be compared without constraints, if structural characterizations are not provided^34^.

Interestingly, we found in our previous studies that the same FA as used in the present study had beneficial effects, such as increased development of larvae and increased protection of the gills of juvenile fish when applied at low to medium concentrations, but exerted detrimental effects at high concentrations^19,35^. Similar results were found with vitamin E (α-tocopherol), which is normally labeled as anti-oxidant but possesses pro-oxidative properties at high concentrations^36^. d'Alençon, et al.^20^ showed that pretreatment of larvae with diphenyleneiodonium (DPI), which inhibits the NADPH oxidase, reduces the number of recruited leucocytes as well, compared to larvae only exposed to copper, showing the involvement of ROS as signaling molecules in the ChIn. The FA has a high phenolic content and a high oxygen scavenging capacity^35^, which might decrease the ROS level inside the larvae directly. However, the number of leucocytes recruited in pretreated DPI larvae in the d'Alençon, et al.^20^ study was still ~ 4 times higher than in copper-untreated larvae, while FA pretreatment decreased the level of ROS to that of control larvae. This shows that although ROS scavenging of the FA might play a minor role, additional anti-inflammatory mechanisms are involved.

We found an increased expression of genes involved in anti-oxidative protection by medium concentrations of FA in our previous study and based on the current results, this could help in preventing the damage of the neuromast and subsequent inflammation by copper. Several studies have shown, that natural compounds, especially polyphenols mediate the transition from pro-inflammatory M1 macrophages to M2 macrophages, which are crucial for inflammation resolution^37–39^. Although we did not find activation of the leucocytes in zebrafish larvae by FA exposure in our previous study^19^, we did find indications towards an increased number of neutrophils and thereby a shift in the composition of innate immune cells. As the used FA is well comparable to polyphenols^35^, it would be an interesting future approach to investigate the overall cell composition, with special emphasis on the different macrophage sub-populations and shifts due to FA exposure.

At high concentrations (300 and 500 mg C/L) we previously found increased expression of anti-oxidative genes as well but combined with high ROS concentrations inside the larvae which caused tissue damage^19^. The same concentrations significantly amplified the copper-induced inflammation in our modified assay. Jiang, et al.^40^ found a strong correlation between the aromatic content and the associated free radicals of HSs and the inflammatory Kashin-Beck disease. Apparently, the phenolic moieties and the persistent free radicals in our FA become hazardous at high concentrations, causing pro-inflammatory effects. This shows that one HSs can exert both, pro- and anti-inflammatory effects and it is essential, to carefully evaluate the effects over a broad range of concentrations and to further compare the effects to the structure of the HS, before declaring any inflammatory properties.

## Conclusion

Low-grade chronic inflammations are promoting numerous age-related diseases and can be caused by many factors including diet, disturbed sleep, physical inactivity, and exposure to pollution^41^. Effective control of inflammation could help to resolve inflammations and protect individuals predisposed to such diseases. At the same time, it is essential to screen for pro- and anti-inflammatory properties of drugs, chemicals, and pollutants to ensure a proper risk assessment. The transparency and short life-cycle of zebrafish larvae allow easy monitoring of in vivo inflammation response, but also to study diseases associated with chronic inflammation. Although without a doubt, the use of GMO zebrafish is of great advantage, it comes with barriers such as the requirement for special governmental licenses and special training and precausions. The manual assay, which can be performed by staining wild-type larvae requires handling and fixation of each larvae individually. Our modification of the ChIn assay allows the use of wild-type zebrafish larvae instead of GMO-strains while allowing for high throughput screening. It can be used as an alternative, when the use of GMOs is inconvenient, or as a complementary method for fast creening of the inflammatory properties of therapeutics which interfer with coppper. HSs can have very diverse chemical building blocks resulting in diverse biological effects between different HSs. Using the modified assay, we demonstrated that the anti-inflammatory effects of a phenol-rich FA turn pro-inflammatory due to the high content of free radicals at higher concentrations. The same chemical properties protecting against oxidative stress and inflammation at low to medium concentrations are responsible for the hazardous pro-inflammatory effects at higher concentrations.

## Material and methods

### Husbandry and general experimental conditions

Parental zebrafish were raised and maintained in the facility of Leibniz-Institute of Freshwater Ecology and Inland Fisheries under recommended conditions (26 °C, 16 h/8 h light/dark cycle, lights on at 6:00 am, 7.5 ± 0.5 mg /L oxygen, pH 7.0 ± 0.3, 120 ± 20 µS/cm, 20.63 mg/L NaHCO_3_ (S3 Chemicals, Germany), 18.75 mg/L sea salt (Tropic Marin, Switzerland), 26.81 mg/L CaCl_2_*2H_2_O (Aquafair, Germany))^19^. Zebrafish were kept in 60 L transparent tanks at group sizes of 30 (1:2 female: male ratio) and fed 3 times daily with commercial flake food (TetraMin Flakes, Tetra, Germany) and 2 times with freshly hatched *Artemia salina* nauplii. Fertilized eggs were collected from parental zebrafish (*Danio rerio*) maintained at our facility under recommended conditions. DIN water (deionized water, 294.0 mg/L CaCl_2_*2H_2_O, 123.3 mg/L MgSO_4_*7H_2_O (Roth, Germany), 63.0 mg/L NaHCO_3_, 5.5 mg/L KCl (Merck Germany), pH 7.0 ± 0.2, DIN EN ISO 15,088) was used as control. Exposure followed the guidelines of the Fish Embryo Acute Toxicity Test OECD 236 (2013). Healthy eggs were transferred individually into 24-well plates with 2 mL exposure solution (DIN water or FA) (sealed with SealPlate sealing film (Roth) and incubated for 96 h (26 ± 0.5 °C, 16 h/8 h light/dark cycle, IPP 110plus (Memmert, Germany)). Basic requirements that were met in all experiments were: overall survival ≥ 90% (control), hatching rate after 96 h ≥ 80% (control), and mortality ≥ 30% in the positive control (4 mg/L 3,4-dichloro-aniline). All larvae spawned naturally and were staged according to Kimmel et al.^44^.

### Detection of reactive oxygen species

The cell-permeant reagent 2’,7’ –dichlorofluorescein diacetate (H2DCFDA Sigma-Aldrich, USA) was used to detect the production of hydroxyl- and peroxyl radicals, and other ROS inside the larvae according to Goody et al.^27^ and Hermann et al.^26^. Briefly, larvae were individually transferred with 100 µL of DIN water into 96-well fluorescence plates (ELISA plate black med. bind. F, Sarstedt, Nürnberg, Germany). Plates were visually controlled using a stereomicroscope (Nikon SMZ 1270, Japan) with a ring light (Slim Ring-light Schott) attached to ensure proper plate assignment with exactly one larva in each well. 100 µL detection solution (1 µg/mL H2DCFDA in DIN water) was added to each well and the fluorescence was detected over a period of 4 h in a plate reader (Infinite M200, Tecan, Switzerland). The fluorescence is directly proportional to the amount of ROS. Wells without larvae were used to detected the background fluorescence of the media. Chemically induced inflammation assay.

### Chemically induced inflammation assay

The chemically induced inflammation assay (ChIn) by d'Alençon et al.^20^ uses copper to induce an inflammatory reaction by damaging the neuromast cells at the lateral line and green fluorescence protein (GFP) labeled zebrafish strains to monitor infiltration of leucocytes. Our approach was to modify this assay to the use of wild-type zebrafish to monitor the anti-inflammatory properties of FA.

#### Copper concentration and time of exposure

To determine a proper working concentration, larvae raised in DIN water were first exposed to different concentrations (0, 10, 25, 50, and 100 µM) of copper (CuSO_4_*5 H_2_O, Sigma-Aldrich) for 2 h at 26.0 ± 0.5 °C. H2DCFDA was added and the emission of fluorescence was detected as described above for 4 h and analyzed after 2, 3, and 4 h. In this first step, we determined the copper concentrations that activate the larvae to produce ROS as well as the period of incubation with the fluorescence dye. In the second step, larvae were exposed to 50 µM and 100 µM of copper for 1 and 2 h, respectively to determine the best copper exposition period. After copper exposure, larvae were washed by transferring them into a crystallizing dish containing at least 130 mL of DIN water before measuring the fluorescence. For each repeat and condition, 12 larvae were used and each test was repeated 3 times independently. Wells without larvae were used to detect background fluorescence.

#### Verification of inflammation assay

To test if the assay described above can be used to determine the anti-inflammatory properties of chemicals, we used two agents, known for their anti-inflammatory properties. Diclofenac and ibuprofen are both non-steroidal antiphlogistics (NSAP) or non-steroidal anti-inflammatory drugs (NSAID), inhibiting the two isoenzymes of cyclooxygenase (COX-1 and COX-2). Both have been shown to behave against physical wounding in zebrafish as predicted^45–47^. Using 1.5 and 3 µM diclofenac, as well as 1 and 10 µM ibuprofen, d'Alençon et al.^20^, were able to significantly reduce the copper-induced infiltration of leucocytes in GFP zebrafish. To verify that copper stimulated the inflammatory ROS production rather than just causing oxidative stress^48^, we incubated 96 hpf larvae with diclofenac (1.5 and 3 µM) and ibuprofen (1 and 10 µM) for 1 h at 26 °C. Afterward, larvae were washed with DIN water and exposed to 50 µM copper (2 h, 26 °C). Production of ROS was detected as described above.

### The inflammatory activity of fulvic acid

The inflammatory activity of FA was analyzed using the assay described above. We used a commercially available fulvic acid (FA, FulvoFeed, HuminTech, Germany) because of its thorough characterization and to exclude effects by contaminations. It is fully water-soluble, with a carbon content of 96.7 g C/L, 88.2% humic-like substances (94.4% FA and 5.6% humic acids), and 29.2% aromatic carbon (of which 7.6% are phenols)^35^. Based on our previous study^19^ larvae were exposed for 96 h to 5, 50, 300, and 500 mg C/L of FA before copper exposure. To minimize chelation of copper with the FA, residues were removed from the larvae surface by transferring larvae into DIN water; subsequently, larvae were exposed to 50 µM copper for 2 h and the production of ROS was measured photometrically as described above. Larvae without FA and copper exposure were used as a negative control, 3 µM diclofenac was used as a positive control (larvae unexposed to FA, 1 h of incubation with diclofenac before copper exposure). A total of 24 larvae was used per group (4 independent repeats with 6 larvae).

### Statistical analysis

The datasets were analyzed using RStudio 1.1.453 software (https://rstudio.com/products/rstudio/download/Libraries: PMCMRplus; dplyr; ggplot2; tidyr; car; openxlsx; tidyverse; gridExtra; grid; pwr; effectsize). Determination of the effect power and sample size are given in the supplement. Data were normalized to average fluorescence values of the control group (no exposure at all) to permit the calculation of fold induction. Differences between means were analyzed by one-way ANOVA with Tukey-HSD post-hoc after verifying assumptions for parametric testing were met (shapiro wilk test and levene test). Differences were considered statistically significant at p < 0.05. Graphs are Tukey boxplots.

### Compliances with ethical standards

Experiments were performed following the European Directive 2010/63/EU of the European Parliament and the Council of the European Union on the protection of animals used for scientific purposes and the German AnimalWelfare Act. Experiments with fish larvae until the start of exogenous feeding do not require special permission by the animal experimental ethics committee of the Berlin State Office for Health and Social Affairs (LaGeSo). All experiments were performed in accordance with the ARRIVE guidelines.

## Supplementary Information

Supplementary Information.

## Data Availability

All data generated or analyzed during this study are included in this published article.
