# Supplementary material for: Modification of the chemically induced inflammation assay reveals the Janus face of a phenol rich fulvic acid
Source: Sci Rep. 2022 Apr 7;12:5886. doi: 10.1038/s41598-022-09782-w (PMC8991211; doi:10.1038/s41598-022-09782-w)
Supplement: Supplementary file 1 — Supplementary Information. [file 41598_2022_9782_MOESM1_ESM.pdf]

Supplements: Sample size determination based on the results of the pilot study

The first part of the modified chemically induced inflammation assay (Chapter 4.3.1 “Copper concentration and time of exposure” in the manuscript) was used as a pilot study to identify the sample size required for the assay.

To determine the effect of the copper exposure against the control, Cohens d [46] against the control group was calculated using the equation

$$d = \frac{mean_2 - mean_1}{SD_{pooled}} = \frac{mean_2 - mean_1}{\sqrt{\frac{sd_1^2 + sd_2^2}{2}}}$$

S1: Calculations of Cohen's coefficient against the control group at different concentrations and exposure periods of HD2DCFDA. Values are rounded to 2 digits

Copper exposure concentration	Mean	SD	Cohens d
**2 h of incubation with the fluorescence dye**			
**0 µM**	1.00	0.10	-
**10 µM**	1.01	0.08	0.11
**25 µM**	1.01	0.09	0.14
**50 µM**	1.00	0.09	0.00
**100 µM**	1.03	0.09	0.32
**3 h of incubation with the fluorescence dye**			
**0 µM**	1.00	0.14	-
**10 µM**	1.01	0.12	0.09
**25 µM**	0.98	0.13	0.17
**50 µM**	1.09	0.11	0.73
**100 µM**	1.12	0.17	0.77
**4 h of incubation with the fluorescence dye**			
**0 µM**	1.00	0.19	-
**10 µM**	1.06	0.13	0.40
**25 µM**	1.07	0.17	0.40
**50 µM**	1.28	0.23	1.35
**100 µM**	1.34	0.28	1.46

The theoretical number of larvae that has to be used to get significant results at different incubation periods with the fluorescence dye (2 h, 3 h, and 4 h) was calculated for larvae exposed to copper using R studio (t-test; Difference between two independent means (two groups)], α=0.05, β=0.2, and β=0.05). In the following simplified presentation, the low concentrations (10 µM and 25 µM copper) were excluded as they did not increase the relative fluorescence significantly until 4 h of exposure.

S2: Sample size required per group when using 2 groups for β =0.2 and β =0.05 after incubation with H2DCFDA.

β =0.2	**Theoretical minimal number of larvae per group when using 2 groups**		
**Copper Concentration**	**After 2h incubation**	**After 3h incubation**	**After 4h incubation**
50 µM	-	31	10
100 µM	155	28	9
β =0.05	**Theoretical minimal number of larvae per group when using 2 groups**		
**Copper Concentration**	**After 2h incubation**	**After 3h incubation**	**After 4h incubation**
50 µM	-	50	15
100 µM	255	45	13

As exposure to 50 µM and 100 µM copper gave similar results, we decided to use the lower concentration, as 100 µM might induce negative effects that are not evident at first. As a compromise between practical feasibility when using more groups and statistical certainty, we furthermore decided to use an incubation time with the dye of 4 h and a sample size of 24 per group for the anti-inflamatory tests (diclofenac and ibuprofen) and the FA.

S3: Two-way ANOVA of copper concentration and incubation time with the fluorescence dye.

	**Df**	**F**	**Pr(>F)**
**Copper exposure concentration**	4	21.75	<2e-16
**H2DCFDA incubation time**	2	39.42	<2e-16

S4: Two-way ANOVA of copper concentration and copper incubation time.

	**Df**	**F**	**Pr(>F)**
**Copper exposure concentration**	1	24.69	1.92e-6
**Copper incubation time**	1	11.11	0.0011
